# Rainbow Trout (*Oncorhynchus mykiss*) Na^+^/H^+^ Exchangers tNhe3a and tNhe3b Display Unique Inhibitory Profiles Dissimilar from Mammalian NHE Isoforms

**DOI:** 10.3390/ijms22042205

**Published:** 2021-02-23

**Authors:** Salvatore Blair, Xiuju Li, Debajyoti Dutta, Danuta Chamot, Larry Fliegel, Greg Goss

**Affiliations:** 1 Department of Biology, Winthrop University, Rock Hill, SC 29733, USA; blairs@winthrop.edu; 2 Department of Biological Sciences, University of Alberta, Edmonton, AB T6G 2E9, Canada; dchamot@ualberta.ca; 3 Department of Biochemistry, University of Alberta, Edmonton, AB T6G 2H7, Canada; xjli@ualberta.ca (X.L.); debajyoti.47@gmail.com (D.D.); lfliegel@ualberta.ca (L.F.)

**Keywords:** AP-1, fish physiology, ion-regulation, pharmacology, pH regulation

## Abstract

Freshwater fishes maintain an internal osmolality of ~300 mOsm, while living in dilute environments ranging from 0 to 50 mOsm. This osmotic challenge is met at least partially, by Na^+^/H^+^ exchangers (NHE) of fish gill and kidney. In this study, we cloned, expressed, and pharmacologically characterized fish-specific Nhes of the commercially important species *Oncorhynchus mykiss*. Trout (t) Nhe3a and Nhe3b isoforms from gill and kidney were expressed and characterized in an NHE-deficient cell line. Western blotting and immunocytochemistry confirmed stable expression of the tagged trout tNhe proteins. To measure NHE activity, a transient acid load was induced in trout tNhe expressing cells and intracellular pH was measured. Both isoforms demonstrated significant activity and recovered from an acute acid load. The effect of the NHE transport inhibitors amiloride, EIPA (5-(N-ethyl-N-isopropyl)-amiloride), phenamil, and DAPI was examined. tNhe3a was inhibited in a dose-dependent manner by amiloride and EIPA and tNhe3a was more sensitive to amiloride than EIPA, unlike mammalian NHE1. tNhe3b was inhibited by high concentrations of amiloride, while even in the presence of high concentrations of EIPA (500 µM), some activity of tNhe3b remained. Phenamil and DAPI were ineffective at inhibiting tNhe activity of either isoform. The current study aids in understanding the pharmacology of fish ion transporters. Both isoforms display inhibitory profiles uniquely different from mammalian NHEs and show resistance to inhibition. Our study allows for more direct interpretation of past, present, and future fish-specific sodium transport studies, with less reliance on mammalian NHE data for interpretation.

## 1. Introduction

Mechanisms allowing for the transmembrane transfer of sodium ions (Na^+^) for hydrogen ions (protons, H^+^) are found universally across various phyla, including bacteria, plants, and animals [1]. Members of the *SLC9* gene family dominate this role in higher vertebrates and these Na^+^/H^+^ exchangers (NHE) extrude one intracellular H^+^ in exchange for one extracellular Na^+^ [2]. In mammals, at least ten functional genes are present that code for the various NHEs (NHE1-NHE10), which are responsible for intracellular pH (pH_i_), cell volume regulation, and transepithelial Na^+^ transport [1,3,4,5,6,7].

Freshwater fishes must maintain an internal osmolality of ~300 mOsm, while living in dilute environments, ranging from 0 to 50 mOsm [8]. This osmotic challenge requires fish to actively absorb the necessary ions (i.e., Na^+^, Cl^−^, etc.) from the environment against the concentration gradient via cellular transport pathways located on the gill epithelium [8,9,10]. Such sodium uptake mechanisms in fishes have long been an important topic and various ion-regulation and osmoregulation strategies take place at the gill [11]. However, many pathways of sodium regulation in the fish gill remain unresolved [8,10,12,13]. The expression of Nhe2, 3a, and 3b isoforms in the gill of freshwater fishes has been demonstrated [14,15,16,17,18,19,20] and all three Nhe isoforms involved in osmoregulation have now been identified in salmonids: Nhe2 (Slc9a2), Nhe3 [Slc9a3 (referred to hereafter as Nhe3a)] [17], and Nhe3b [21]. However, the physiological properties and pharmacological inhibitor profiles have not been well characterized. In fishes, recent investigations have suggested that Nhe3b serves as a principal mechanism for Na^+^ uptake and H^+^ excretion at the gill [22,23], while Nhe3a is primarily expressed in the kidney [17,20].

Pharmacological inhibitors that block the action of an ion regulatory proteins are a useful method used to demonstrate the presence or function of a specific ion channel or transporter in physiological studies [24,25,26]. Traditionally compounds, such as amiloride (MK 870; N-amidino-3,5-diamino-6-chloropyrazinecarboxamide), have been used. Amiloride is a diuretic in humans, and is an inhibitor of human NHE isoforms [26,27]. Counillon and colleagues (1993) demonstrated K_i_ values (concentration of drug that results in half the maximum inhibition) for amiloride on human NHE1, NHE2, and NHE3 expressed in NHE deficient cell lines as 3 μM, 3 μM, and 100 μM, respectively, with NHE3 the most resistant to amiloride inhibition. This pattern was the same for the other NHE inhibitors including 5-N, N-dimethyl amiloride (DMA), 5-N-methyl-propyl amiloride (MPA), and (3-methylsulphonyl-4-piperidinobenzoyl (HOE694), with NHE1 being most sensitive, followed by NHE2, and NHE3 demonstrating highest resistance. The K_i_ values for HOE694 were 0.16, 5, and 650 μM for NHE1, NHE2, and NHE3, respectively. The K_i_ values for the modified amiloride derivative MPA for the three isoforms were 0.08, 0.5 and 10 μM, respectively. EIPA (5-(N-ethyl-N-isopropyl)-amiloride) is another commonly used amiloride derivative modified similarly to MPA, and its K_i_ for NHE1, NHE2, and NHE3 are 0.3, 1.8, and 67 μM, respectively [28,29].

To date, almost all interpretations from fish specific experiments in vivo utilizing inhibitory drugs, have been based on the known pharmacological profiles in mammalian NHEs [26] with an overall assumption of applicability of species crossover. However, the applicability of these compounds to fishes has not been well established, and while pharmacological agents have been employed to investigate the modes of Na^+^ acquisition [25,30,31,32,33,34], there has been little study of direct effects on the protein, and profiles for these agents have not been confirmed for fish Nhes directly. Multiple Na^+^ transport pathways are known to exist in gill ionocytes making results from pharmacological inhibition studies difficult to accurately interpret (see reviews [10,35,36]).

A complete characterization of pharmacological inhibitor profiles in a system without the interfering effects of the possible multiple Na^+^ transport pathways present in the fish gill will allow for more precise interpretation of fish Nhe and Na^+^ transport pharmacology. The aim of this study was to clone and characterize fish-specific Nhe3a and Nhe3b and examine drug inhibitor profiles by expression of rainbow trout transporters in an NHE-deficient cell system. This would allow for direct pharmacological characterization of each isoform independently. We tested the effects of Amiloride, EIPA, (a derivative of amiloride more potent in inhibition of mammalian NHE’s), DAPI, and Phenamil [37], which inhibits epithelial sodium channels and has been used to study sodium uptake earlier [38,39]. Our results demonstrate that trout tNhe3a and tNhe3b are active, can be studied in the NHE-deficient system, and that they have unique inhibitory efficacy, different from that of the mammalian NHE. 

## 2. Results

### 2.1. Cloning and Analysis of Nhe3a and Nhe3b

We successfully cloned *nhe3a* and *nhe3b* from *O. mykiss* as described above. Sequence analysis of the cDNA’s confirmed that they were identical to database entries NM_001130995 and FJ376630.1 for *nhe3a* and *nhe3b,* respectively. A comparison of the deduced amino acid from these sequences with human NHE1 and NHE3, zebrafish *nhe3a* and *nhe3b*, and *Squalus nhe3* is shown in Supplementary Figure S1. Trout *nhe3a* and nhe3b have 58 and 56% identity with human NHE3 protein, respectively, and 35 and 35% identity with human NHE1 protein, respectively. *Oncorhynchus mykiss nhe3a* was 67% identical with zebrafish *nhe3a* and *O. mykiss* nhe3b was 64% identical with zebrafish *nhe3b* (Table S2). The cytosolic regulatory membrane domain of human NHE1 is approximately 315 amino acids and begins at amino acid 500 [40]. From this region on, the amino acid identity with trout nhes decreases relative to that in the membrane domain. *Oncorhynchus mykiss nhe3b* has a longer amino acid sequence than *O. mykiss nhe3a* with more regions of identity to other nhe isoforms. 

### 2.2. Tissue Gene Expression

**Figure 1:**
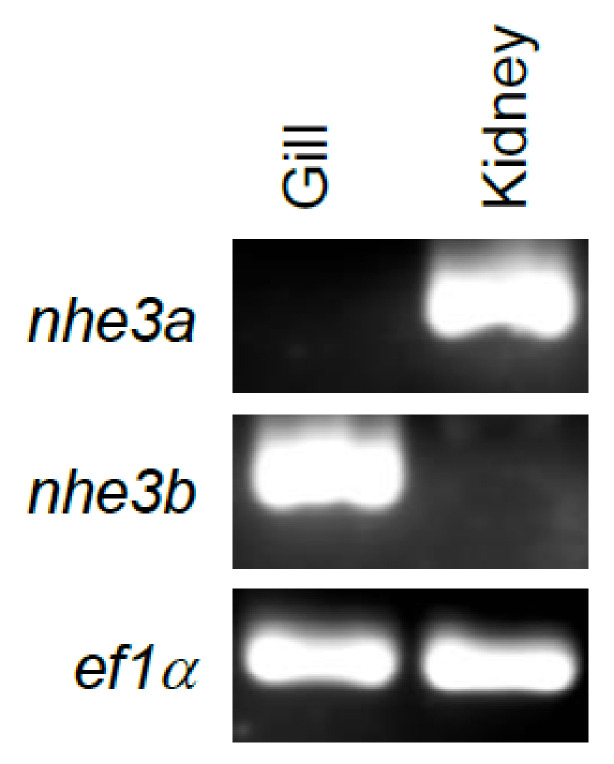
RT-PCR analysis of gene expression in trout tissues. Total RNA was extracted from adult rainbow trout gill and kidney tissue and analyzed with RT-PCR using gene specific primers for *nhe3a nhe3b*, and *ef1α (elongation factor 1 alpha)*.

Gene expression analysis with RT-PCR revealed that *O. mykiss nhe3a* and *nhe3b* were tissue specific. Expression of *nhe3a* was localized to the kidney, with no appreciable *nhe3b* expression detected in the kidney, while *nhe3b* was localized to the gill as shown by a single PCR product, with no appreciable expression of *nhe3a* in the gill (Figure 1).

### 2.3. Transfection and Protein Expression

**Figure 2:**
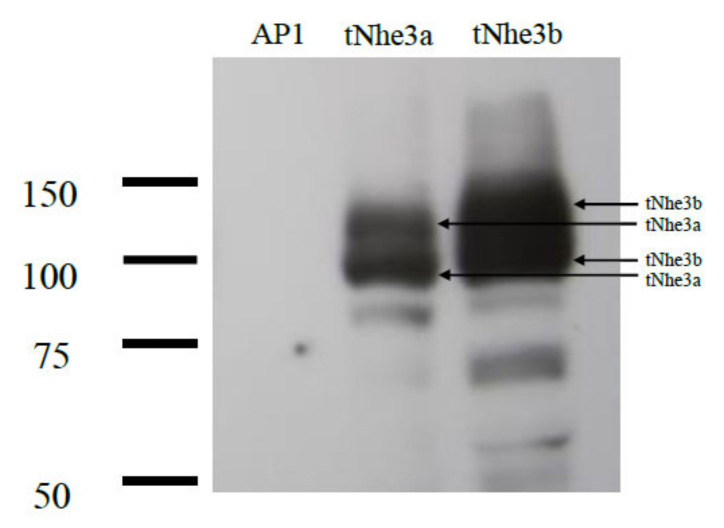
Western blot analysis of expression tNhe3 proteins. Western blot of whole cell lysates of stable cell lines expressing tNhe3a or tNhe3b proteins. Moreover, 100 μg of total protein was loaded in each lane. The sample was immunoblotted with anti-GFP tag antibody. AP-1 is a cell lysate from mock transfected AP-1 cells.

Transfection of AP-1 cells with the trout Nhe (tNhe) constructs resulted in stable colonies that showed resistance to G418 selection. We screened colonies for expression of fusion protein using anti-GFP (tag) antibodies. We obtained positive GFP expression at appropriate molecular weights in each cell line demonstrating expression of tNhe3a and tNhe3b (Figure 2). Trout Nhe3a was slightly smaller than tNhe3b as predicted from its molecular weight. Trout Nhe3a was mainly present as two bands at ~100 and ~115-kDa band with some evidence of aggregation. Trout Nhe3b was also mainly present as two bands, one approximately 105 kDa and a second about 125 kDa.

**Figure 3:**
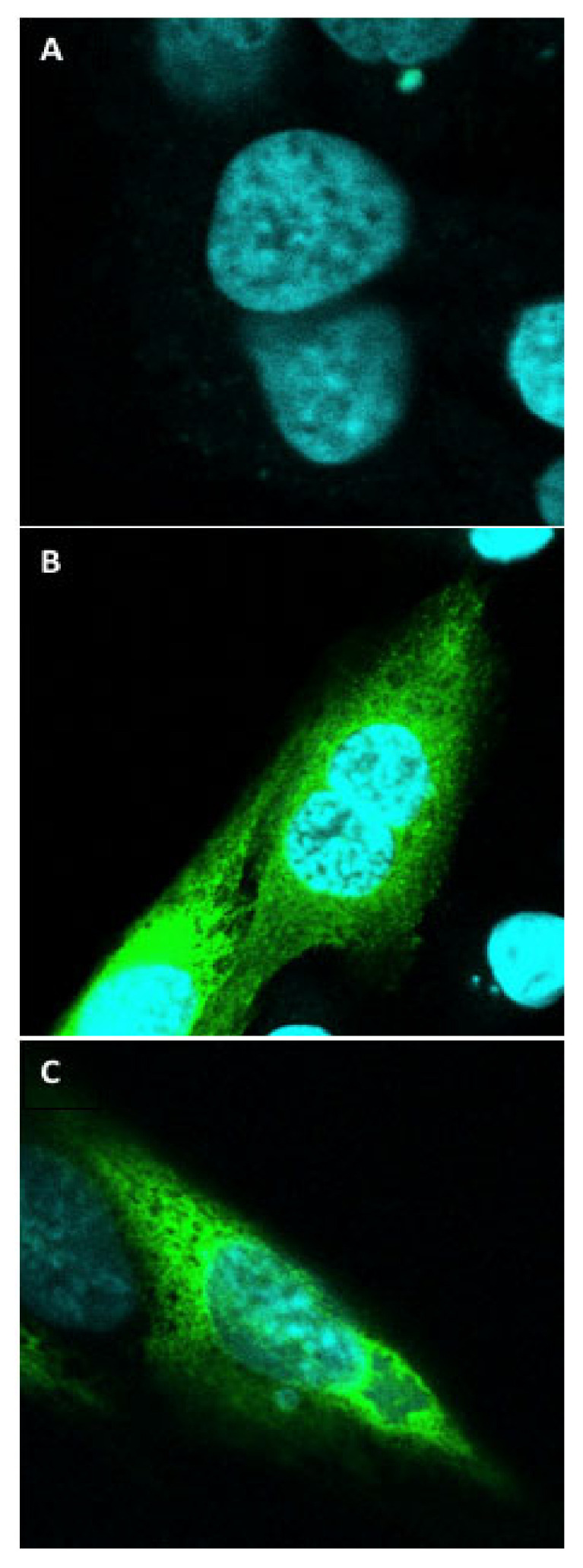
Confocal imaging of expression tNhe3 proteins. Confocal fluorescent imaging (63 × objective lens) of cell preparations of stable AP-1 cell lines either non-transfected or expressing tNhe3a or tNhe3b proteins. (**A**) Non-transfected AP-1 cell stained with DAPI. (**B**) GFP tagged-tNhe3a expressing cells. (**C**) GFP tagged-tNhe3b expressing cells. Intracellular and cell membrane protein expression is present in each of the stably expressing tNhe cell lines. Scale bar represents 20 μm.

Confocal microscopy analysis indicated prominent expression of both tNhe3a and tNhe3b in their respective stably transfected cells (Figure 3). Expression of tNhe3a and tNhe3b was fairly ubiquitous throughout, localized to the plasma membrane as well as intracellularly.

### 2.4. tNHE Activity

**Figure 4:**
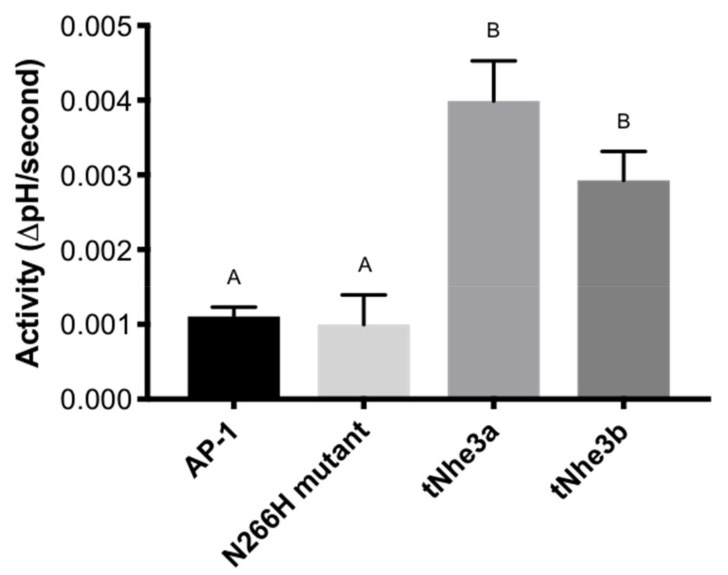
Summary of activity of tNhe3 proteins in stably transfected AP-1 cells. Activity was measured after ammonium chloride pre-pulse as described in the “Materials and Methods”. Results are ∆ intracellular pH/s. Data are presented mean ± SE, while dissimilar letters indicate statistical significance between groups as demonstrated by one-way ANOVA, with Tukey’s multiple comparisons test (n ≥ 6, *p* < 0.05, ANOVA).

Measurement of Na^+^/H^+^ exchanger activity using the ammonium chloride prepulse method demonstrated that NHE activity was 3–4 fold greater in both tNhe3a and tNhe3b expressing cells than in the NHE-deficient AP-1 cells and the NHE1 defective N266H mutant control cells (Figure 4). While tNhe3a appeared to show slightly more activity than tNhe3b, the difference was not significant and was not corrected for differences in expression levels. The background rate of recovery of AP-1 cells (equivalent to that of the NHE1 defective mutant) was subtracted from all pharmacological analysis.

### 2.5. Pharmacological Inhibition

**Figure 5:**
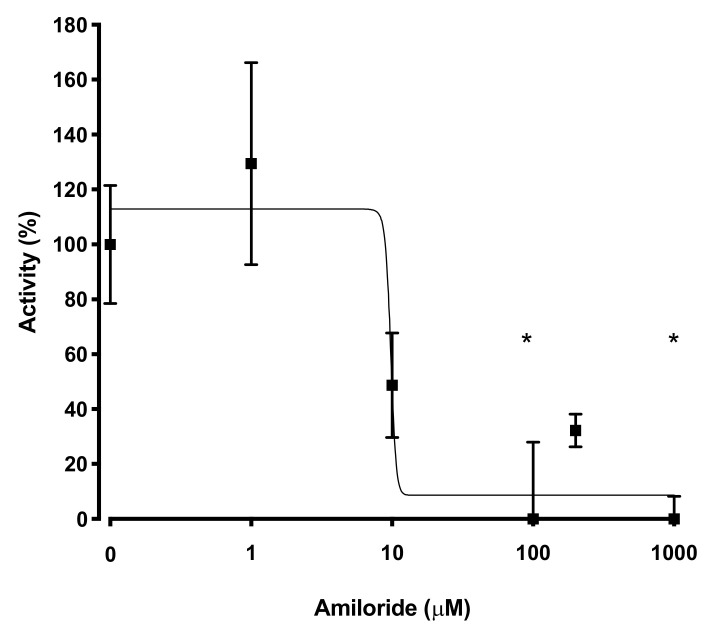
Effect of varying concentrations of amiloride on activity of tNhe3a. tNhe3a containing cells were subjected to a two pulse Na^+^/H^+^ exchanger activity assay and the activity (ROR, rate of recovery) of the exchanger in the second pulse was compared to that of the first pulse. The second pulse was in the presence of the indicated inhibitor. A control was in the presence of equal amounts of vehicle (DMSO). Amiloride concentrations were from 1 to 1000 µM. IC50 was estimated at 9.3 uM as described earlier [28]. Raw data presented in Figure S2. Asterisk * indicates significantly different from the control at *p* < 0.05, one-way ANOVA, with Tukey’s multiple comparison’s test. Data are presented as mean +/− SE. Each data point is mean of 8–11 measurements.

**Figure 6:**
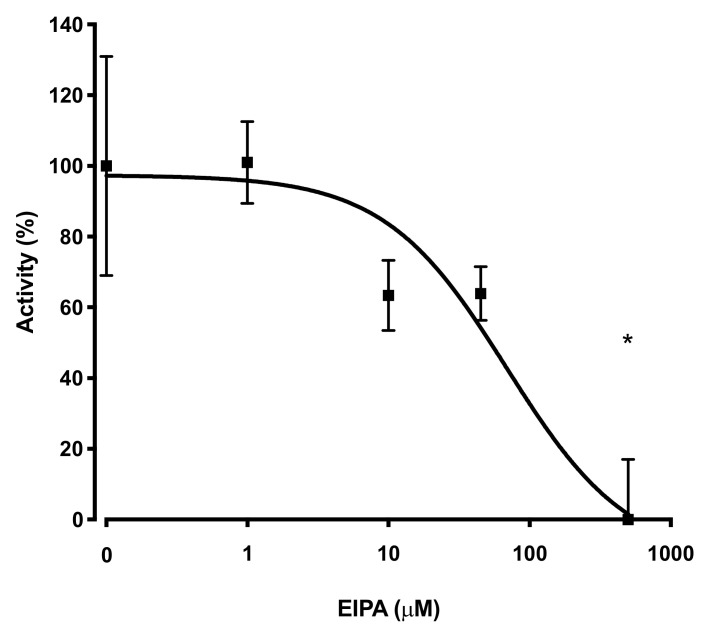
Effect of varying concentrations of EIPA on activity of tNhe3a. tNhe3a containing cells were subjected to a two pulse Na^+^/H^+^ exchanger activity assay and measured as described in Figure 5. EIPA concentrations were from 1 to 500 µM. IC50 estimated at 44 µM. Raw data presented in Figure S3. Asterisk * indicates significantly different from the control at *p* < 0.05, one-way ANOVA, with Tukey’s multiple comparison’s test. Data are presented as mean +/− SE and each experimental point is 8–10 measurements, control n = 18.

**Figure 7:**
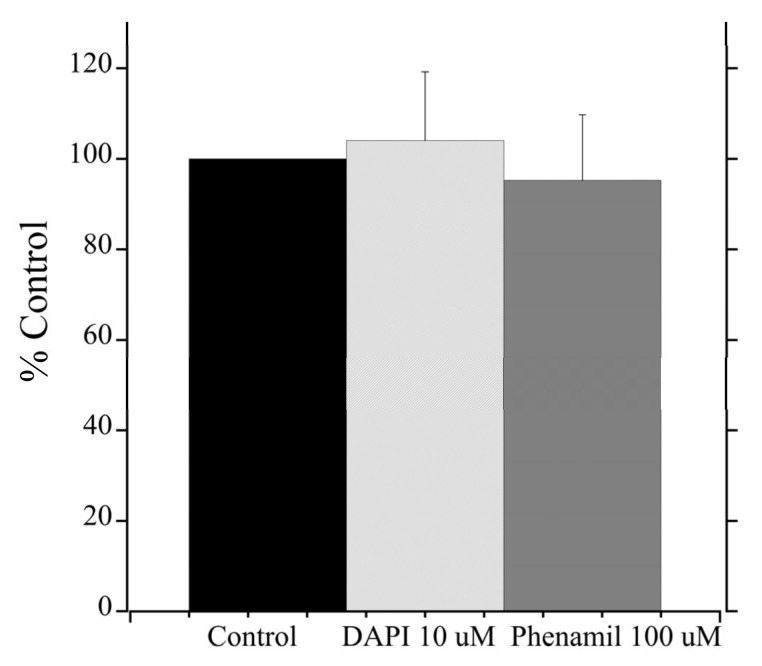
Effect of phenamil, or DAPI on tNhe3b activity. The cells were subjected to a two pulse Na^+^/H^+^ exchanger activity assay and measured as described in Figure 5. The control treatment was in the presence of equal amounts of vehicle (DMSO). Na^+^/H^+^ exchanger activity presented as % of control AP1 cells. Asterisk ^*^ indicates significance from the control as demonstrated by one-way ANOVA at *p* < 0.05. Results are mean +/− SE of at least eight independent experiments.

**Figure 8:**
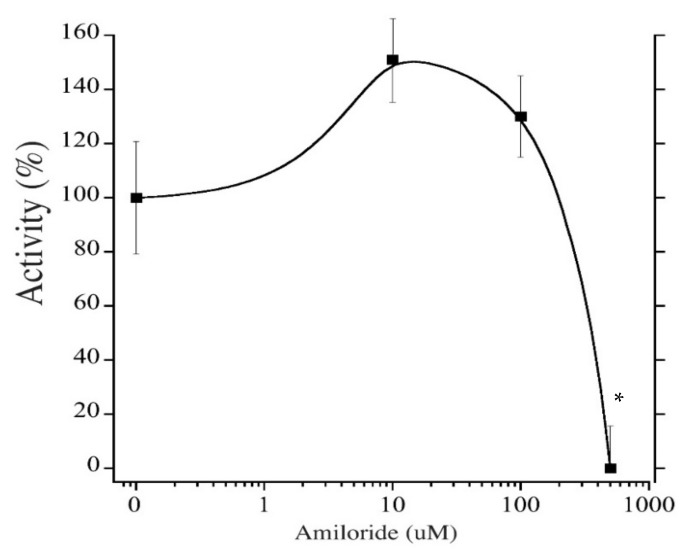
Effect of Amiloride on tNhe3b activity. The cells were subjected to a two pulse Na^+^/H^+^ exchanger activity assay and measured as described in Figure 5. A control was in the presence of equal amounts of vehicle (DMSO). Asterisk * indicates significantly different from the control at *p* < 0.05, one-way ANOVA, with Tukey’s multiple comparison’s test. IC50 relatively incalculable, but approximately 335 µM. Each experimental point is mean +/− SE of 7–11 measurements, control n = 17.

**Figure 9:**
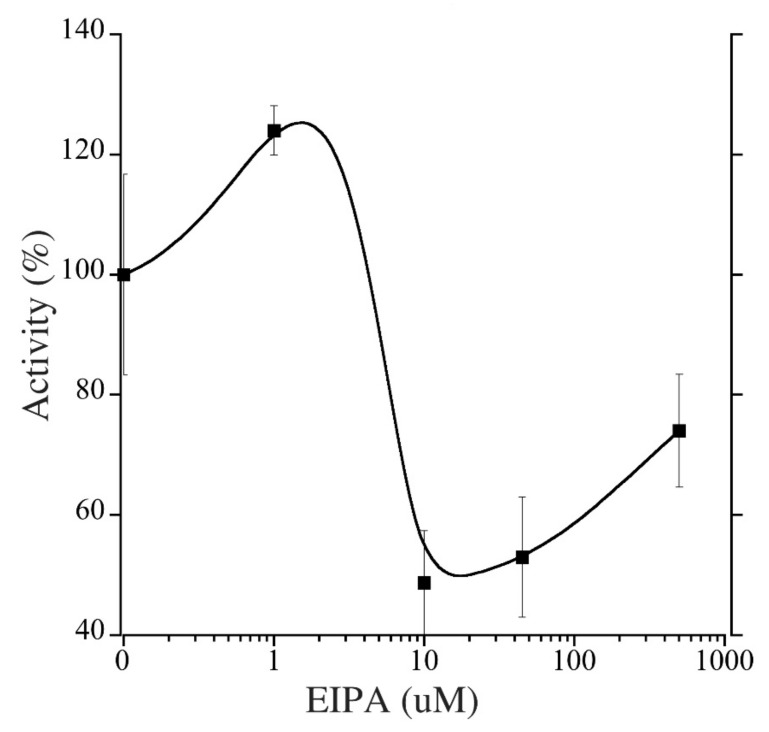
Effect of varying concentrations of EIPA on activity of tNhe3b. tNhe3b containing cells were subjected to a two pulse Na^+^/H^+^ exchanger activity assay and the activity was measured as described in Figure 5. EIPA concentrations were from 1 to 500 uM. IC50 cannot be calculated. Results are mean +/− SE of 8–16 independent experiments with no significant differences (*p* < 0.05, ANOVA).

To estimate the efficacy of various inhibitors in inhibition of tNhe3a activity, we subjected the tNhe3 expressing clonal lines to varying doses of several common NHE inhibitors. Amiloride was a potent inhibitor of tNhe3a activity with a dose-dependent decrease in activity demonstrated and an estimated IC_50_ of 9 μM (Figure 5). Similar to amiloride, tNhe3a activity was also inhibited by EIPA in a dose-dependent manner (Figure 6). Estimation of IC_50_ was 44 μM. There was no effect of 100 μM phenamil and 10 μM DAPI, two commonly employed doses previously demonstrated to reduce Na^+^ uptake in rainbow trout, on tNhe3b activity (Figure 7). For tNhe3b activity, amiloride at either 10 or 100 μM did not significantly reduce activity; only at 500 μM did amiloride significantly reduce tNhe3b activity (Figure 8). In contrast, EIPA reduced the activity of tNhe3b, but EIPA even at higher doses (500 μM) was unable to inhibit more than 40–50% of control activity (Figure 9). It was not possible to calculate an IC50 for the inhibition. 

## 3. Methods

### 3.1. Animals 

Rainbow trout, *Oncorhynchus mykiss,* were raised from embryos generously donated from Allison Creek Brood Trout Hatchery, Coleman, Alberta. Embryos were maintained in Heath trays with aerated 10 °C flowing dechlorinated Edmonton city tap water until hatch, and then maintained in the Biological Sciences animal sciences facility until use. All animal use was approved under University of Alberta animal use protocol number AUP00000072. All trout were euthanized in buffered MS222 (1 g/L MS222, 2 g/L NaHCO_3_) and filaments from right and left gill arches, along with kidney tissue, were excised from three adult fish, immediately frozen in liquid nitrogen, and stored at −80 °C for RNA isolation. 

### 3.2. RNA Isolation, cDNA Synthesis, and PCR

RNA isolation, cDNA synthesis, PCR and initial cloning strategies were followed as described previously [41]. RNA extraction (TRIzol Reagent, Ambion, Life Technologies, Carlsbad, CA, USA) from frozen gill and kidney tissues (50–100 mg) from three rainbow trout was performed as per the manufacturer’s protocol. In each tissue sample, the RNA pellet was resuspended in 50 μL of nuclease-free water and RNA checked for quality and purity using both NanoDrop, ND-1000 (Thermo Fisher Scientific, Waltham, MA, USA) and formaldehyde RNA gel electrophoresis. A total of 10 μg of RNA was then treated for DNA contamination with Recombinant DNase I (Ambion, Austin, TX, USA) and 2 μg of DNase-treated RNA used in the subsequent 1st strand complimentary DNA (cDNA) synthesis by SuperScript III Reverse Transcriptase (Invitrogen, Carlsbad, CA, USA) with a mix of random and oligo(dT) primers as per manufacturer’s protocol. 

Reverse transcription PCR (3 min denaturation at 95 °C; and 35 cycles of 95 °C for 30 s, and 30 s at 56 °C (*nhe3a*, *nhe3b*, *ef1α*), and 72 °C for 1 min, final elongation at 72 °C for 5 min) was performed on RNA extracted from tissues (gill and kidney) of three adult *O. mykiss* and visualized by electrophoresis on a 2% agarose gel stained with ethidium bromide and imaged using AlphaImager 2200 (ProteinSimple, San Jose, CA, USA). The identities of all PCR products were confirmed by sequencing.

### 3.3. Cloning

For *O. mykiss nhe3a*, gene specific primers containing Sma1 and Sal1 restriction sites (Table S1) were designed and direct amplification with Phusion High-Fidelity polymerase (NEB, Ipswich, MA, USA) from *O. mykiss* kidney cDNA resulted in a ~2.2 kbp fragment. The PCR product was cleaned up with QIAquick PCR Purification Kit (Qiagen, Hilden, Germany) and ligated into Sma1 and Sal1 digested pDisplay plasmid vector (Thermo Fisher Scientific) using T4 DNA Ligase (NEB, Ipswich, MA, USA). Chemically competent *Escherichia coli* DH5α cells (Thermo Fisher Scientific, Waltham, MA, USA) were transformed with the ligated plasmid containing the *O. mykiss nhe3a* insert. Colonies were grown overnight on Lysogeny broth (LB) Ampicillin (100 μg/mL) plates and underwent blue–white screening. Positive colonies were picked and cultured in 5 mL of LB-Amp media at 37 °C overnight. Colony PCR was performed on selected bacterial colonies from each of the plates containing individual transformed bacteria. Plasmids were then isolated from bacterial cultures using GeneJET Plasmid Miniprep Kit (Thermo Fisher Scientific, Waltham, MA, USA). Restriction digest of resulting plasmid DNA and sequencing was performed to confirm presence of the specific *nhe* in the pDisplay vector. Identity of the product was confirmed by sequencing and alignment using Clustal Omega software. 

Due to initial complications with amplifying the *nhe3b* gene using restriction sites incorporated directly into initial primers for insertion into pDisplay, prior cloning into pBluescript plasmid vector (Agilent, Santa Clara, CA, USA) was performed. For *nhe3b,* full-length gene primers were designed (Table S1) and amplification of *O. mykiss nhe3b* (from gill cDNA) PCR product with Phusion High-Fidelity polymerase (NEB, Ipswich, MA, USA) resulted in a ~2.8 kbp amplicon. PCR products were cleaned as before, inserted into pBluescript SK cut with EcoRV restriction enzyme, and blunt end ligation was completed using T4 DNA Ligase (NEB, Ipswich, MA, USA). Identical transformation and plasmid isolation steps were taken as above with the *nhe3b* insert in the pBluescript vector. Restriction digest of resulting plasmid DNA along with sequencing was performed to confirm presence of *nhe3b* in the pBluescript vector. A second set of primers containing Sma1 and Sal1 restriction sites (Table S1) were designed against *O. mykiss nhe3b* and PCR amplification was run using the resulting plasmid DNA as a template. Following PCR clean up, the resulting PCR products were then ligated into pDisplay Vector, which were previously digested with Sma1 and Sal1 restriction enzymes. The procedure for transforming *E. coli* bacterial competent cells and isolating plasmid DNA was repeated with this ligation product. The resulting plasmid DNAs (pDisplay containing the *nhe3a* and *nhe3b* inserts) were used as templates for sub-cloning into the plasmid pmEmeraldDectin1A-N-10-GFP (kindly provided by Dr. N. Touret, Department of Biochemistry, University of Alberta), which has an enhanced Green Fluorescent Protein GFP C-terminal to the protein Dectin. Dectin was removed with a Nhe1 to Age1 digestion and PCR products of the *O. mykiss* cDNA clones were amplified with a third set of primers (Table S1) to insert the products in frame with GFP. PCR products contained an upstream Nhe1 site and a downstream in frame XmaI or Age1 that were used for cloning. After cloning, all constructs were sequenced (at the Alberta Proteomics and Mass Spectrometry Facility) to ensure the fidelity of amplification. 

### 3.4. Cell Culture and Stable Transfection

To characterize the activity of the wild type vs. mutant Na^+^/H^+^ exchanger, trout tNhe3a, and tNhe3b constructs were stably transfected (LIPOFECTAMINE™ 2000, Thermo Fisher Scientific, Waltham, MA, USA) into an AP1 cell line which is a derived Chinese hamster ovarian cell line that does not express their own NHE protein [42,43]. The plasmids contain a neomycin resistance marker that allows for stable selection of transfected cells using G418 antibiotic. Cell lines were regularly re-established from frozen stocks between passage numbers 5 and 11 and cultured in alpha-MEM medium supplemented with 10% Fetal Bovine Serum, and 25 mM HEPES, pH 7.4. Transfected and non-transfected AP1 cells were grown under incubation conditions of 5% CO_2_ at 37 °C. Results are typical of at least two stable cell lines made independently and cultured in the presence of the G418 antibiotic to ensure selection stable transfection with tNhe constructs. 

### 3.5. SDS-PAGE and Immunoblotting

To confirm the expression of *t*Nhe3a and tNhe3b in stably transformed AP1 cell lines, we performed immunoblotting against the GFP tag of the NHE proteins. Samples of cell lysates were made as described earlier [44], and 100 μg of protein (Bio-Rad D/C^TM^ Protein Assay, Hercules, CA, USA) was separated on SDS-PAGE (10%) gels, transferred to nitrocellulose, probed with 1° anti-GFP polyclonal Ab (rabbit-anti GFP, 1:1000, a gift from Dr. L. Berthiaume, Department of Cell Biology, University of Alberta) and 2° Ab (goat-anti rabbit-HRP, 1:1000, Jackson ImmunoResearch, West Grove, PA, USA) and visualized using X-ray film via the Amersham enhanced chemiluminescence western blotting and detection system. 

### 3.6. Immunocytochemistry

To further confirm and localize the expression of *O. mykiss* tNhe3a and tNhe3b in stably transformed AP-1 cell lines, immunocytochemistry was performed. Transfected (tNhe3a and tNhe3b) and non-transfected AP-1 cells were grown to 70% confluency on glass coverslips. Cells were washed in phosphate buffered saline solution (PBS), pH 7.4, and then fixed in 4% paraformaldehyde for 10 min and washed 3 times in 1X PBS. Staining with DAPI (300 nM) was conducted for 10 min in the dark followed by two rinses with PBS and one with water. Coverslips were then mounted with DAKO fluorescent mounting medium (Agilent, Santa Clara, CA, USA). Images were obtained with a Leica SP5 confocal laser scanning microscope with a 63X objective. Lasers and laser intensity were 405 Diode for DAPI, 10% detection at 435–465 nm. Argon for GFP, 20% at 510–560 nM. Images were obtained using Leica Application Suite Advanced Fluorescence software (Leica Microsystems, Wetzlar Germany) and processed identically.

### 3.7. Intracellular pH Measurement (Na^+^/H^+^ Exchange Activity)

To measure pH_i_, cells were grown to approximately 80–90% confluence on glass coverslips, incubated for 20 min at 37 °C with BCECF-AM (1.875 μg/mL) as described earlier [45], and fluorescence measured using a PTI Deltascan spectrofluorometer (Photon Technology International, Birmingham, NJ, USA). Na^+^/H^+^ exchanger activity was measured after an acute acid load was induced as described earlier [45]. Ammonium chloride (50 mmol/L × 3 min) addition followed by removal was used to induce the acute acidosis and the first 20 s of recovery in NaCl-containing medium (135 mM NaCl, 5 mM KCl, 1.8 mM CaCl_2_, 1 mM MgCl_2_, 5.5 mM glucose, and 10 mM HEPES, pH = 7.4) was measured as ΔpH/s. This rate of recovery after acute acidosis was measured in the wild type AP-1 cells (deficient of NHE) and compared with the rate of recovery in the *O. mykiss t*Nhe3a and tNhe3b transfected AP-1 cells. As an additional transfection control to validate that the intracellular pH_i_ recovery seen were indeed a function of Na^+^/H^+^ exchange activity, the previously described null activity N266H mutant NHE1 protein cell line [46], was measured for activity. To test the effects of various inhibitors on *O. mykiss t*Nhe3a and Nhe3b activity, a dual pulse method was used. We have previously shown that the second pulse in the dual pulse assay is equivalent to the first [28]. Cells were acidified with ammonium chloride in the absence of inhibitor (but with vehicle of 0.1% DMSO) and allowed to recover. This was followed by a second pulse, which was the same except that recovery was in the presence of the indicated concentration of inhibitor, therefore, each population served as its own control. Calibration of pH_i_ fluorescence was done for each cell population using nigericin as described earlier [44]. All assay solutions were kept at 37 °C immediately prior to coming in contact with cells. For pharmacological analysis, the background rate of recovery of AP-1 cells (equivalent to that of the NHE1 inactive mutant) was subtracted from all values in tNhe3a and tNhe3b containing cells. Due to the nature of the activity assays and rates of activity, any final calculations producing negative numbers signifying complete inhibition of activity were portrayed as zero on subsequent graphs. Results are shown as the mean ± S.E. of at least six independent cellular measurements. All raw data were checked for normality with Shapiro–Wilk test, log transformed if necessary, and analyzed by one-way ANOVA with Tukey’s multiple comparisons post-hoc test relative to control DMSO value with *p* < 0.05 as significant (GraphPad Prism Software, San Diego, CA, USA).

## 4. Discussion

In this study, we examined the isolated and cloned *nhe3a* and *nhe3b* isoforms of the trout Na^+^/H^+^ exchanger. Cloning and expression of the proteins was successful as demonstrated by the presence of western blotting of the appropriate sized, GFP-tagged proteins (Figure 2). Both clones were near their predicted weight with the GFP tag (tNhe3a- 111 kDa; tNhe3b- 121 kDa) though membrane proteins often run at anomalous molecular weights on SDS-PAGE [47]. The presence of two bands representing tNhe3a and tNhe3b is noteworthy. The smaller bands may represent degradation, truncated proteins, or alternatively, a different level of glycosylation. Human NHE1 is typically present in this expression system as two bands, one a fully glycosylated protein and one with reduced levels of glycosylation [43,44]. 

Further confirmation of protein expression in these stable cell lines was indicated with detection of the GFP-tagged tNhe3a and tNhe3b proteins using confocal microscopy. The prominent fluorescence indicated clear evidence of transfection and targeted exogenous expression of the tagged trout transporters. While plasma membrane localization is visible, a significant amount of intercellular expression also occurred in both of the transfected stable cell lines. Nevertheless, the unequivocal functional protein expression of each trout Nhe isoform is indicated by the activity assays demonstrating significantly higher Na^+^/H^+^ exchange activity in cells transfected with tNhe3a and tNhe3b, compared with the non-transfected AP-1 cells or the mutated NHE inactive (N266H) cell line. To our knowledge, this is the first direct demonstration and measurement of activity of these proteins. Relative to the earlier demonstrated activity of mammalian NHE1 protein [46,48,49,50], the activity was lower. The likely explanation is that the protein was not well targeted to the cell surface, as shown by our localization experiments. The mammalian NHE1 C-terminal, tail has several regions that are important in targeting the protein to the cell surface and when these are absent, targeting to the cell surface is reduced [51]. These targeting sequences are not present in the tNhe3a and tNhe3b isoforms. Additionally, depending on the cell type, NHE3 can be present in significant intracellular compartments and may have more of a predisposition to an intracellular localization compared with NHE1 [52,53,54]. The absence of mammalian cell surfacing targeting signals, along with the known predisposition of NHE3 to form intracellular pools, likely explains the relatively high amount of intracellular NHE3 and the lower activity of the protein that was observed.

For both trout isoforms, we observed unique and unusual effects in our testing of putative inhibitory compounds. For tNhe3a, amiloride was about four times more potent than EIPA. This is the opposite result seen in mammalian Na^+^/H^+^ exchangers where EIPA has been reported to be as much as 200X’s more potent than amiloride [55]. Additionally, EIPA has been reported to have an IC50 for mammalian NHE1 of around 0.02 μM, and for NHE3 of 2–8 μM [56], while our result was about 40 μM. Our results with amiloride and tNHE3a are more similar to those observed in the mammalian Na^+^/H^+^ exchangers. The IC50 for amiloride for NHE1 and NHE3 has been reported to be 3 μM for NHE1 and 40–100 μM for NHE3 [56] while we found an IC50 of 9 μM. This unusual reversal of potencies of amiloride and EIPA is, to our knowledge, a rare finding. Not only did it occur with tNhe3a, it also occurred with tNhe3b, which was even more resistant to inhibition. Concentrations of 500 μM were required to inhibit activity and for EIPA, even 500 μM resulted in only partial inhibition of activity. In this regard, the effect of EIPA on tNhe3b was similar to that of the effect on tNhe3a, being a less effective inhibitor than amiloride.

Aside from the present study, a previous report also used a similar approach, transfecting mammalian NHE-deficient cells with *Tribolodon hakonensis* (Redfin dace) Nhe3. They also found that EIPA resistance was greater than that of human NHE1 and NHE3 [16]. However, a different study examined the sensitivity of Zebrafish (*Danio rerio*) Nhe3b and found that this protein was more sensitive to EIPA than amiloride [57]. Our research group also performed a pharmacological analysis on Nhe2 and Nhe3 from *Squalus suckleyi* (Dogfish) using the same assay as in the present study, providing IC_50_ values for amiloride and EIPA (55 µM and 4.8 µM, respectively) on dfNhe2, as well as for dfNhe3 of (24 µM and 9 µM, respectively) [28]. In this case, EIPA was more potent than amiloride, similar to the mammalian proteins. We have demonstrated that *nhe3b* isoform was expressed in the trout gill, while *nhe3a* was localized in the trout kidney. The expression of *nhe3a* and *nhe3b* in the trout kidney and gill, respectively, aligns with previous observations of two *nhe3* isoforms expressed in trout tissues [17,18,20].

That tNhe3a and tNhe3b are relatively resistant to inhibition by amiloride and EIPA may be explained by their changes in their amino acid composition compared with mammalian NHEs. Trout Nhe3a and Nhe3b were more homologous to the drug resistant human NHE3 than to the sensitive NHE1 protein. The membrane domain, responsible for cation transport, is approximately 500 amino acids in human NHE1, which corresponds approximately to amino acids 475 and 455 for trout Nhe3a and Nhe3b. Na^+^/H^+^ exchangers are thought to have a “Na^+^/H^+^ exchanger fold”, which is critical in ion transport. Within the membrane domain of human NHE1, two transmembrane segments TMIV and TMXI (amino acids 153–177 and 449–470, respectively) are thought to form this fold [58]. These transmembrane segments are also both thought to be involved in inhibitor binding. For TMIV of human NHE1, the sequence 161-FFLFL-165 is critical. The corresponding sequence in partially inhibitor resistant mammalian NHE2 is -FFLYL- and in more resistant NHE3 it is FFFYL [59]. Changing the NHE2 protein to -FFLYL- or -FFFFL- increases amiloride resistance about 5- and 10-fold, respectively. A similar observation was that mutation of (the hamster amino acid residue) equivalent to human NHE1 Leu163 to Phe, also causes a large increase in the IC_50_ for amiloride [60]. In both trout, Nhe3a and Nhe3b, this sequence is FFFYL (Supplementary Figure S1) that is equivalent to inhibitor resistant human NHE3. Thus, the sequence of this region may partially account for the large resistance to inhibition that was shown by trout Nhes. Other studies showed that mutation of Gly174 of human NHE1 to Asp, causes a large increase in amiloride resistance [6,61]. This residue remains as Gly in all NHE isoforms shown (Supplementary Figure S1), except interestingly, it is a Ser in zebrafish Nhe3a and Nhe3b. 

It should be noted, however, that it is likely that the binding site of amiloride and other inhibitors have contributions from several transmembrane segments that form some kind of inhibitor binding pocket [4]. In this regard, TMXI has also been implicated in both inhibitor sensitivity and NHE1 transport activity [58,62], though specific amino acids that affect inhibitor efficacy are not well delineated. The residues of *O. mykiss* equivalent to TMXI of human NHE1 (449–470) are 423 and 444 of tNhe3a and 403–424 of tNhe3b (Figure 1). This segment of *O. mykiss* is also well conserved, showing more conservation with human NHE3 proteins than with NHE1. It is interesting to note that Squalus *suckleyi* has the sequence FFFYLL intact with IC50 values of 9 and 24 uM for amiloride and EIPA, respectively [28]. These values are quite different from those of *O. mykiss* reported here supporting the suggestion that other contributions from transmembrane segments form part of the inhibitor binding pocket. In this regard, we have recently modeled the NHE1 protein and inhibitor docking analysis suggested two binding sites of inhibitors that included contributions from amino acids of extracellular loops 4, 5, and 6 and transmembrane segments 2, 3, 4, 5, 6, 8, and 11 [63]. Exact amino acids binding at each site varied depending on the inhibitor. It is possible that for EIPA and the tNhe3b protein, the incomplete inhibition found in our present study can be explained by the absence of one of these two sites. Future studies could examine this possibility.

Relatively high concentrations of phenamil (100 µM) and DAPI (10 µM) used in the present study do not inhibit tNhe3b, while the same or lower concentrations significantly block sodium uptake in whole animal studies [64,65]. Lower concentrations of phenamil (10 µM) and DAPI (1 µM) were also tested with no effect on activity (data not shown). Our results showing the lack of inhibition by phenamil and DAPI, agree with studies demonstrating that phenamil is a potent sodium channel inhibitor with little effect on NHE function while DAPI is an inhibitor of the acid sensing ion channel [26,65,66]. It should also be noted that these studies applied inhibitors for periods of one hour or greater in whole animals, which could result in changes to the level of NHE protein expressed or targeted to the cell membrane, or changes to regulatory proteins, while our study examined acute effects on the proteins over periods of less than one minute. 

Although the expression of NHE isoforms has been localized in various freshwater fish species including *Oncorhynchus mykiss* [14,17,18,20,21], freshwater adapted *Fundulus heteroclitus* [67], *Oreochromis mossambicus* [68], *Danio rerio* [69], *Oryzias latipes* [12], and *Tribolodon hakonensis* [16], NHEs are only one of multiple modes of sodium uptake. When studying freshwater fish physiology, it is of interest to know which pathway of Na^+^ transport is involved in a particular species and environment. Other pathways of sodium uptake include an epithelial acid sensing ion channel (ASIC) [65], or other apical transporters such as the Na^+^/Cl^−^ cotransporter (NCC) [19,70]. Our study demonstrates that amiloride can be useful for inhibition of tNhe3a, but may be of questionable use to study tNhe3b. The concentrations we used in the present study were equal to or exceeded the concentration levels resulting in maximum sodium transport inhibition observed in previous whole-animal fish studies (i.e., 500 µM amiloride, 100 µM EIPA, 1µM DAPI, 50 µM Phenamil [65]; 100 µM amiloride [71,72]). 

Physiological research innovation requires applying experimental methods and techniques in a cross disciplinary and cross-species manner (i.e., utilizing mammalian based pharmacological inhibitors in fish physiological studies). However, given the existing complexity of sodium transport systems in freshwater fishes, relying solely on mammalian pharmacological inhibitor profiles to understand cross species transporter characteristics may lead to spurious interpretations. Thus, achieving fish specific inhibitor profiles for cellular transporters aids in clarifying these intricate pathways. 

Limitations to this effort do exist to our study and we acknowledge the following caveats for the current study. Rainbow trout are poikilothermic cold-water fish, living in waters with an optimal temperature of <20 °C [73]. Thus, trout cellular functions are optimized to this temperature and following transfection of trout *nhe* into AP-1 cells, the 37 °C cell culture environment may result in altered protein pharmacology. However, it was demonstrated earlier that reducing the temperature from 37 °C to 25 °C had little effect on cariporide (an NHE inhibitor) potency in mammalian cells [74]. A different limitation is that the relatively high external sodium concentrations used in the experimental assay solutions (135 mM NaCl), may change the relative IC_50_ found in freshwater fish studies with lower Na^+^ concentrations, because of interactions between the Na^+^ transport site and the inhibitor binding site [75].

Overall, the results of our study indicate vast differences in pharmacological sensitivities that are Nhe-isoform-dependent and species-dependent. The results suggest that the tNhe3 proteins have unique inhibitor binding sites, compared with the human proteins. Additionally, we demonstrate an unusual inhibitory profile with amiloride more potently inhibiting activity than EIPA in the tNhe3 protein. These data should be considered when interpreting future applications involving fish Nhe transport. Additionally, the drug inhibition data will be useful in interpretation of future fish sodium transport experiments and limit the need to rely solely on relevant mammalian transporter pharmacological profile literature.

## Data Availability

The data presented in this study are available on request from the corresponding author.

## Supplementary Materials

The following are available online at https://www.mdpi.com/1422-0067/22/4/2205/s1, Figure S1: Alignment of NHE proteins, Figure S2: Raw data points associated with Figure 5 (Effect of varying concentrations of amiloride on activity of tNhe3a), Figure S3: Raw data points associated with Figure 6 (Effect of varying concentrations of amiloride on activity of tNhe3a), Table S1: Sequences of primers and gene accession numbers used for cloning nhe3a and nhe3b from rainbow trout, Oncorhynchus mykiss, Table S2: Percent identity of trout amino acid sequences Nhe3a and trout Nhe3b with other NHEs.
