# Supplementary material for: Rainbow Trout (Oncorhynchus mykiss) Na+/H+ Exchangers tNhe3a and tNhe3b Display Unique Inhibitory Profiles Dissimilar from Mammalian NHE Isoforms
Source: Int J Mol Sci. 2021 Feb 23;22(4):2205. doi: 10.3390/ijms22042205 (PMC7926675; doi:10.3390/ijms22042205)
Supplement: Supplementary file 1 [file ijms-22-02205-s001.pdf]

10 Supplemental Figures

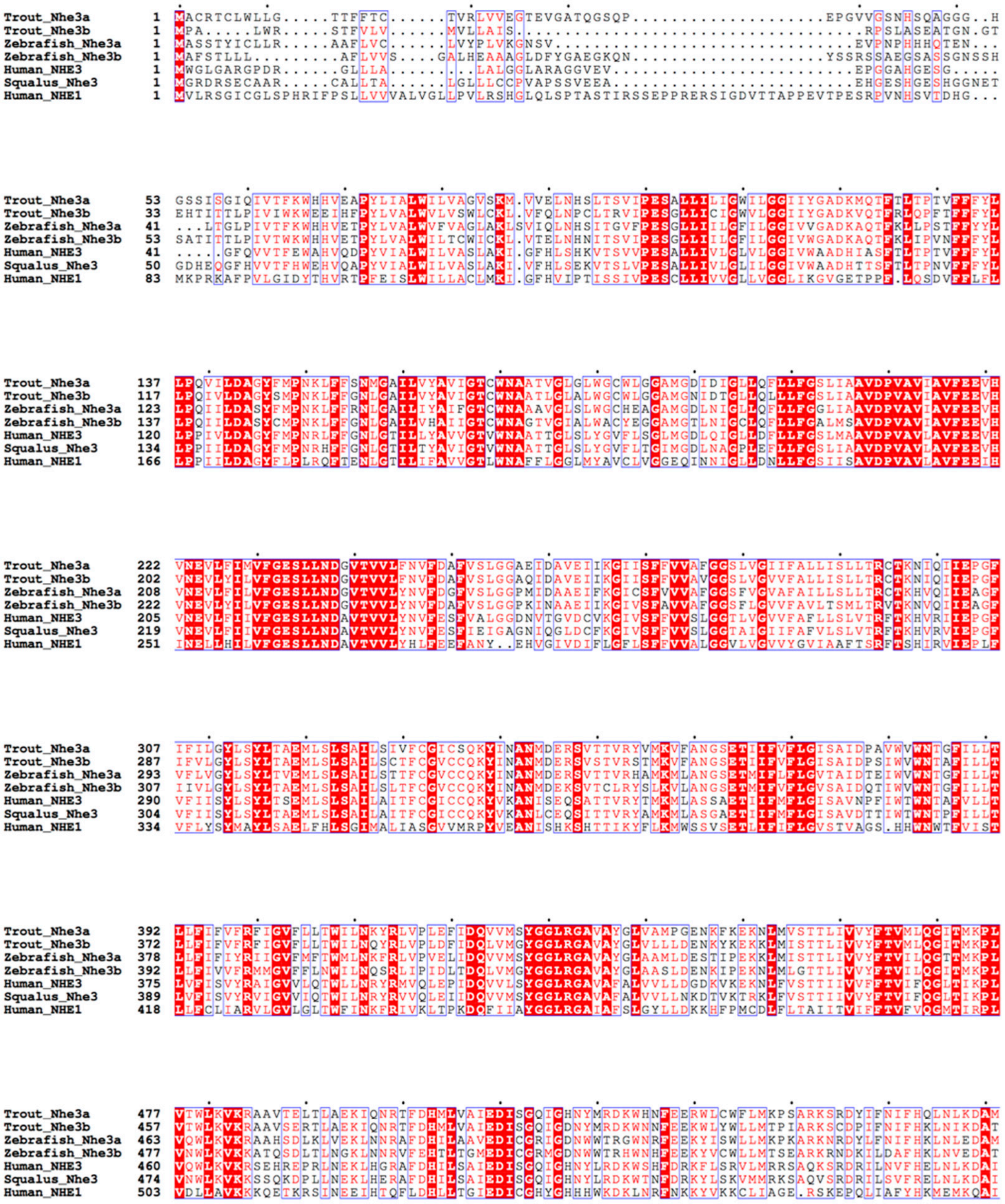

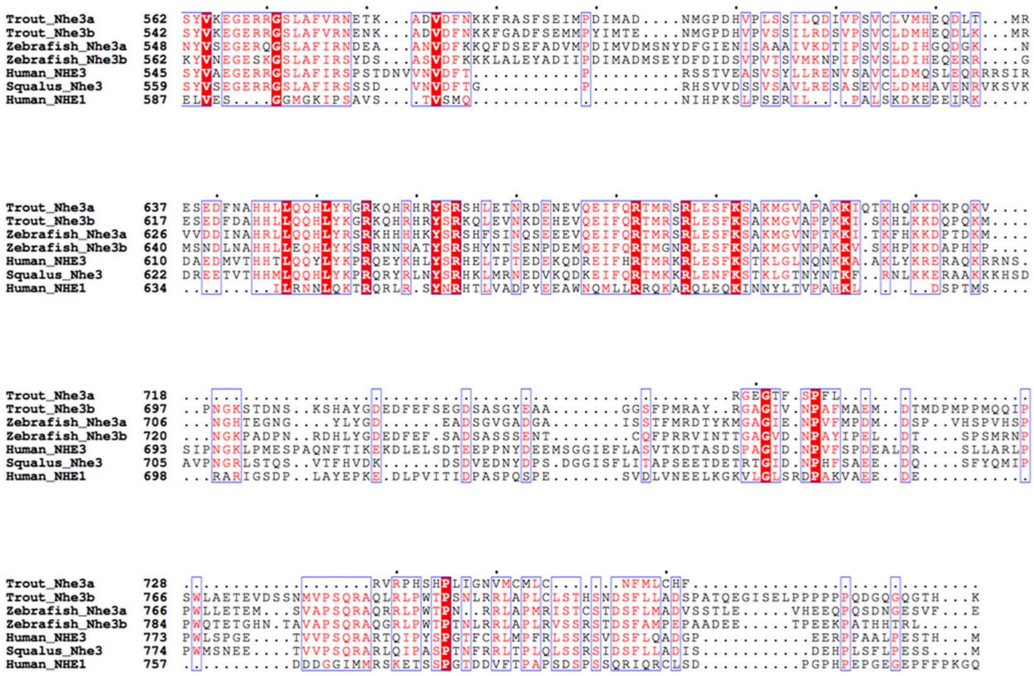

Supplemental Figure 1. Alignment of NHE proteins. Alignment of trout, zebrafish, human NHE1, and NHE1 and Squalus NHE3 protein. Conserved amino acids are colored red and conserved regions are boxed. White amino acids boxed in red represent regions of identity. Representatives included are; *Oncorhynchus mykiss*, Nhe3a, NM_001130995; Nhe3b, FJ376630.1; *Danio rerio*, Nhe3a, EF591984.1; Nhe3B, EF591980.1; *Homo sapiens*, NHE3, NG_046804.1; *Squalus suckleyi*, Nhe3, KR297236.1; *Homo sapiens*, NHE1, NM_003047. Amino acid alignment was using the MAFFT program [75].

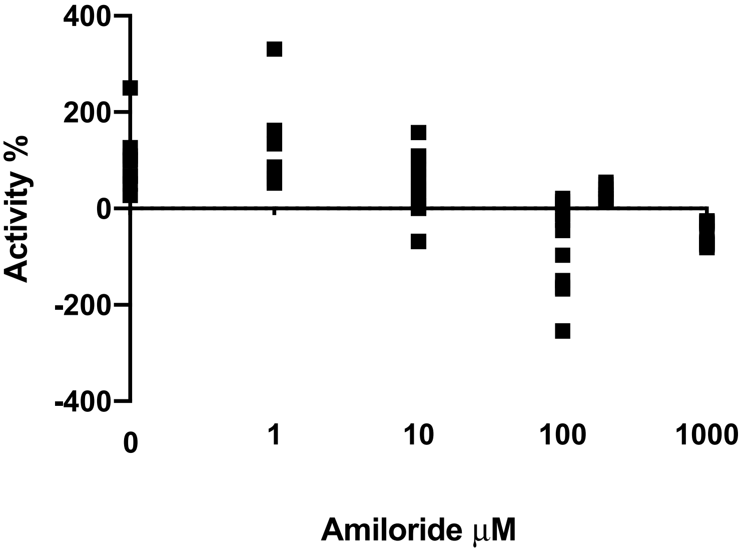

Supplemental Figure 2. Raw data points associated with Figure 5 (Effect of varying concentrations of amiloride on activity of tNhe3a). Each individual data point is relative to the mean of the control (DMSO) which was normalized to 100% activity.

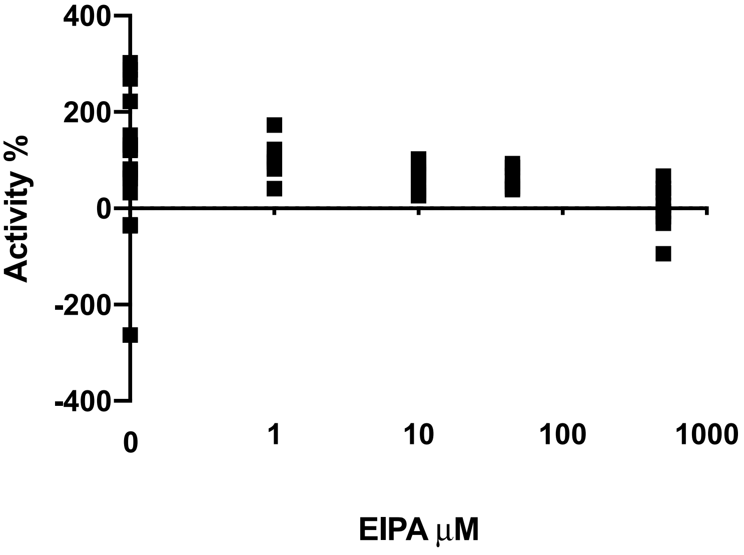

Supplemental Figure 3. Raw data points associated with Figure 6 (Effect of varying concentrations of amiloride on activity of tNhe3a). Each individual data point is relative to the mean of the control (DMSO) which was normalized to 100% activity.

Supplemental Tables

Supplemental Table S1. Sequences of primers and gene accession numbers used for cloning *nhe3a* and *nhe3b* from rainbow trout, *Oncorhynchus mykiss*. Restriction enzyme sites used in cloning are underlined.

Gene	Primer Set	Accession Number	Primer	Primer Sequence (5′– 3′)
nhe3a	1st set	NM_001130995.1	Forward	TACCCGGGATGGCATGCAGAACTTGC
			Reverse	CGCGTCGACAAAATGACATAACATGAAATTACAC
nhe3a	2nd set	NM_001130995.1	Forward	AAGAATTCGCTAGCGCCACCATGGCATGCAGAACTTGCCTCTG
			Reverse	CCGGAATTCACCGGTCCAAAATGACATAACATGAAATTACA
nhe3b	1st set	NM_001160482.1	Forward	CCAAGATTGATCAAAGCTCAAG
			Reverse	AAAGAGGTCAGTTTGTGGA
nhe3b	2nd set	NM_001160482.1	Forward	TACCCGGGATGCCAGCTCTG
			Reverse	CGTGTCGACCTTGTGGGTTCC
nhe3b	3rd set	NM_001160482.1	Forward	AAGAATTCGCTAGCGCCACCATGCCAGCTCTGTGGCGCTCCACTTTCG
			Reverse	CCGGAATTCGACCGGTCCCTTGTGGGTTCCCTGACCCTGTCCGTC

Supplemental Table S2. Percent identity of trout amino acid sequences Nhe3a and trout Nhe3b with other NHEs.

	Trout_Nhe3a	Trout_Nhe3b	Zebrafish_Nhe3a	Zebrafish_Nhe3b	Human_NHE3	Squalus_Nhe3	Human_NHE1
Trout_Nhe3a	100.00	78.77	66.44	61.80	57.66	55.48	35.19
Trout_Nhe3b		100.00	64.37	63.95	55.67	53.62	35.05
Zebrafish_Nhe3a			100.00	65.41	53.46	52.62	33.60
Zebrafish_Nhe3b				100.00	48.75	48.52	33.85
Human_NHE3					100.00	73.35	38.40
Squalus_Nhe3						100.00	38.52
Human_NHE1							100.00
